# Comparison of Intralesional Triamcinolone Acetonide, 5-Fluorouracil, and Their Combination in Treatment of Keloids

**Published:** 2018-05

**Authors:** Sunil Srivastava, Aditya Patil, Chaitra Prakash, Hiranmayi Kumari

**Affiliations:** Sawai Man Singh Medical College and Hospital, Jaipur, India

**Keywords:** Keloid, Triamcinolone, Fluorouracil

## Abstract

**BACKGROUND:**

Despite the myriad options available, there is no universally accepted treatment for keloids. This study has compared intralesional triamcinolone acetonide, 5-fluorouracil, and their combination in treatment of keloids.

**METHODS:**

In this randomized parallel group study, 60 patients were enrolled and randomly allocated to three groups. Patients received intralesional injections of triamcinolone acetonide (TAC) in Group TAC, 5-fluorouracil (5FU) in Group 5FU and a combination in Group T+F every 3 weeks till 24 weeks or till the keloid resolved.

**RESULTS:**

There was a reduction in all parameters at every successive assessment in all three groups. Improvement in terms of height, vascularity and pliability was fastest with 5FU, TAC and T+F group, respectively, which was statistically significant. Decrease in pigmentation was significantly faster with T+F. Reduction in pruritus, however, was significantly faster with 5FU than the other groups, but the difference in reduction of pain among the three groups was not significant. Telangiectasias and skin atrophy were seen most commonly in TAC group, while skin ulceration was a common problem in 5FU group.

**CONCLUSION:**

TAC, 5FU and their combination are all effective in keloid scars. A combination of TAC+5FU seems to offer the balanced benefit of faster and more efficacious response with lesser adverse effects when compared to individual drugs.

## INTRODUCTION

Keloids occur as a result of abnormal wound healing. The exact cause for this disorder remains elusive despite ongoing research and hypotheses. Keloids are known for a lack of standardized treatment and a high propensity for recurrence. This is evident in the wide range of available treatment modalities like surgical excision, cryotherapy, laser therapy, low-dose radiation, silicone sheeting, topical retinoids and intralesional injections of steroid, 5-flurouracil (5FU) and bleomycin being employed.^3^ All of these regimens are empirical, none of which guarantee a definite cure.

Triamcinolone acetonide (TAC), a long-acting glucocorticoid has been the most popular drug and can presently be considered as gold standard in keloid treatment, alone or in combination.^3^^-^^5^ A clinical efficacy ranging from 50-100% and a recurrence rate ranging between 9% and 50% has been reported., 5-flurouracil (5FU), a pyrimidine analogue, was first introduced in the treatment of keloid by Fitzpatrick who published his results in 1999. Since then, several studies have documented its efficacy in keloids. 5FU has a comparatively faster response in scar flattening. Combining TAC with 5FU has been suggested to have a rapid response in terms of scar flattening with an added advantage of fewer side effects., Studies have compared this combination with other drugs and modalities, but these studies cannot be directly compared to each other owing to inconstant outcome variables. To the best of our knowledge, there is no study that simultaneously compares these two drugs alone and as a combination. This randomized study was undertaken with the objective of comparing these three regimens viz. triamcinolone alone, 5-fluorouracil alone and a combination of triamcinolone and 5-fluorouracil in terms of subjective and objective outcomes, and adverse effects. A short review and results of the study are discussed.

## MATERIALS AND METHODS

This was a single-blind, randomized parallel group study conducted in the Department of Plastic Surgery, Sawai Man Singh Medical College and Hospital, Jaipur, India. Patients were enrolled between January 2016 and June 2016 from the out-patient clinic. The study protocol conformed to the guidelines of the Declaration of Helsinki and was approved by the institutional Ethics Review Committee. Informed consent was obtained from all the participants.

Inclusion criteria included patients aged 18 to 60 years with keloids of size 1 to 10 cm in greatest dimension and of >6 months duration. Keloids were diagnosed on the basis of history and clinical examination. Hypertrophic scars were not included. Exclusion criteria were females who were pregnant or were planning pregnancy, patients who had received treatment for keloids in the past 12 months, those who had active inflammation, infection or ulcer in or around the keloid, immunosuppressed patients, patients with chronic inflammatory diseases, renal or liver failure. Complete blood count, renal and liver function tests were done before inclusion and repeated at the fourth visit and last visit. 

Detailed history and demographic parameters were recorded, including etiology and region of keloid. Etiology was broadly divided into spontaneous, infective and traumatic. A total of 60 patients were enrolled for the study and were randomly allocated to one of three groups using a computer-generated random sequence. A single contiguous keloid per patient was considered for the study. Keloids in Group TAC received intralesional triamcinolone acetonide (TAC) 40 mg/ml, keloids in Group 5FU received intralesional 5-fluorouracil (5FU) 50 mg/ml and those in Group T+F received intralesional injection of a combination of TAC (40mg/ml) and 5FU (50mg/ml) in a ratio of 1:9. The drugs used were undiluted except for the said combination.

Injections were made with 27G insulin syringe such that volume injected did not exceed 0.5 ml per square centimeter of keloid. Whenever necessary, multiple pricks were made 1 cm apart to ensure complete and uniform distribution. A maximum of 2 ml was injected per session. Injections were administered every 3 weeks till 24 weeks or till the keloid resolved, whichever was earlier. No local infiltration of anesthetics was done; analgesic was administered orally. Patients received no other therapies like scar massage, laser therapy or pressure garments during the course of study.

All patients were evaluated prior to every injection and a final evaluation was performed 30 weeks after first dose. Evaluations were done by two independent observers who were blinded to the treatment groups. Evaluation was done objectively using Vancouver Scar Scale (VSS) and subjectively by assessing pain and pruritus. Adverse effects at the time of injection and other complaints during the course of treatment were also recorded. 

VSS was originally designed by Sullivan et al to assess burn scars which has since been extended to include other scars as well,. For VSS, keloid height was measured with calipers; pliability was assessed by palpation; vascularity was assessed by visual inspection; pigmentation was scored after blanching and comparing it with the surrounding skin. Blanching was achieved using a piece of clear plastic sheet. Pain and pruritus were scored on a 3-point scale as follows: 0=no pain/pruritus; 1=mild; 2=moderate; 3=severe pain/pruritus. 

Comparative survival analysis between the three groups was done using Kaplan Meier curves to compare rate of improvement. Wilcoxon test was used to compare survival distribution among groups with the test statistics based on differences in group mean scores. Pain and pruritus scores were compared between the three groups using chi square test for qualitative analysis and ANOVA for difference in means of groups. Statistical analysis was carried out with SPSS software for Windows Version 23.0 (Armonk, NY). A *p*-value of <0.05 was considered to be significant.

## RESULTS

A total of 60 patients completed the study. The youngest patient included in the study was 18 years old and the eldest was 56 years old. There were 26 males and 34 females in the study. Infective etiology (n=37) was the commonest etiology followed by traumatic (n=16) and spontaneous (n=7). Pre-sternal region (n=32) was the most frequently involved region, followed by trunk (n=14), extremities (n=10) and face (n=4). The baseline characteristics in terms of age, sex, etiology and region involved were comparable in all three groups (Table 1). 

**Table 1:** Baseline Characteristics of patients

**Variable**	**TAC**	**5FU**	**T+F**	***p*** ** value** *
Age (mean±SD), (years)	26.35±6.11	27.55±8.54	29.9±10.19	0.41
Sex, Number (%)				
Female	12 (60)	10 (50)	12 (60)	0.76
Male	8 (40)	10 (50)	8 (40)	
Etiology, Number (%)				
Infective	12 (60)	11 (55)	14 (70)	0.64
Traumatic	6 (30)	5 (25)	5 (25)	
Spontaneous	2 (10)	4 (20)	1 (5)	
Region, Number (%)				
Presternal	11 (55)	12 (60)	9 (45)	0.69
Trunk	5 (25)	2 (10)	7 (35)	
Extremities	3 (15)	4 (20)	3 (15)	
Face	1 (5)	2 (10)	1 (5)	

* All *p* values were greater than 0.05 (no statistically significant difference).

**Fig. 1:**
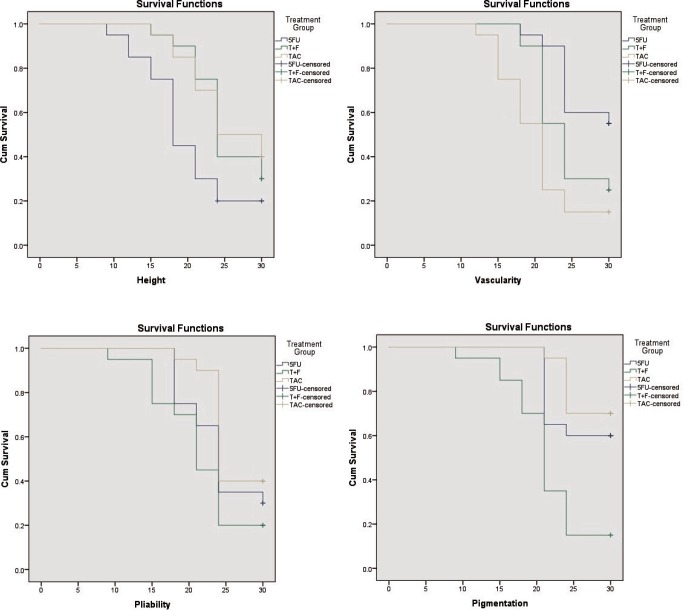
Kaplan Meir survival curves for height, vascularity, pliability and pigmentation

There was no significant difference in baseline pre-injection scores of pain and pruritus and all four parameters of VSS (Table 2). Mean pre-injection VSS scores for all treatment groups at every evaluation are presented in Table 3. There was a reduction in height, vascularity, pliability and pigmentation at every successive assessment in all three groups which was maintained till the final evaluation. The rate of improvement can be seen in the Kaplan Meir curves (Figure 1). 5FU had the lowest survival curves for height, TAC for vascularity and T+F for both pliability and pigmentation. Statistically significant differences among groups in terms of reduction of vascularity and pliability were noted after 6^th^ week while that of height and pigmentation were noted after 3^rd^ week (Table 3).

**Table 2:** Baseline scores of outcome parameters

**Variable**	**TAC**	**5FU**	**T+F**	***p*** ** value** *
Height	1.7±0.57	1.8±0.41	1.9±0.31	0.368
Vascularity	1.85±0.37	1.9±0.31	2±0	0.226
Pliability	2.8±0.41	2.65±0.49	2.8±0.41	0.463
Pigmentation	1.85±0.37	1.8±0.41	1.85±0.37	0.892
Pruritus	2.75±0.44	2.85±0.37	2.7±0.47	0.535
Pain	2.05±0.89	2.3±0.86	2.55±0.69	0.163

† Data reported as Mean±SD.

* All *p* values were greater than 0.05 (no statistically significant difference).

**Table 3:** Mean pre-injection VSS scores

**VSS parameters**	**Group**	**Mean Pre-injection VSS Scores** *									
		**0w**	**3w**	**6w**	**9w**	**12w**	**15w**	**18w**	**21w**	**24w**	**30w**
Height	TAC	1.7±0.57	1.7±0.57	1.65±0.49	1.65±0.50	1.6±0.50	1.4±0.60	1.2±0.70	0.75±0.55	0.65±0.51	0.61±0.45
	5FU	1.8±0.41	1.8±0.41	1.65±0.49	1.25±0.55	1±0.56	0.85±0.59	0.45±0.51	0.3±0.47	0.2±0.41	0.2±0.41
	T+F	1.9±0.31	1.9±0.31	1.9±0.31	1.8±0.41	1.4±0.50	1.05±0.39	0.9±0.31	0.7±0.47	0.35±0.49	0.3±0.47
Vascularity	TAC	1.85±0.37	1.85±0.37	1.75±0.44	1.4±0.50	1.05±0.39	0.75±0.44	0.55±0.51	0.25±0.44	0.15±0.37	0.15±0.37
	5FU	1.9±0.31	1.9±0.31	1.9±0.31	1.9±0.31	1.7±0.47	1.2±0.41	0.95±0.22	0.9±0.31	0.55±0.51	0.55±0.51
	T+F	2±0	2±0	2±0	1.55±0.51	1.1±0.31	1±0	0.9±0.31	0.5±0.51	0.25±0.44	0.25±0.44
Pliability	TAC	2.8±0.41	2.8±0.41	2.6±0.50	2.2±0.52	1.85±0.49	1.6±0.50	1.2±0.52	1.05±0.51	0.8±0.52	0.75±0.44
	5FU	2.65±0.49	2.65±0.49	2.45±0.51	2.1±0.55	1.6±0.50	1.4±0.50	1.1±0.64	0.65±0.49	0.5±0.51	0.5±0.51
	T+F	2.8±0.41	2.65±0.49	2.05±0.69	1.6±0.75	1.2±0.52	1±0.73	0.8±0.62	0.45±0.51	0.35±0.49	0.35±0.49
Pigmentation	TAC	1.85±0.37	1.85±0.37	1.85±0.37	1.8±0.41	1.65±0.49	1.4±0.5	1.05±0.22	1±0.32	0.7±0.47	0.7±0.47
	5FU	1.8±0.41	1.8±0.41	1.8±0.41	1.75±0.44	1.35±0.49	1.1±0.31	1±0	0.65±0.49	0.6±0.50	0.6±0.50
	T+F	1.85±0.37	1.85±0.37	1.7±0.47	1.35±0.59	0.95±0.22	0.85±0.37	0.7±0.47	0.35±0.49	0.15±0.37	0.15±0.37

* Values denoted as Mean±SD.

Comparison using Wilcoxon test showed that improvement in terms of height, vascularity and pliability was fastest with 5FU, TAC and T+F group respectively which was statistically significant. Decrease in pigmentation was seen faster with T+F than with individual drugs alone, which was highly significant. Pain and pruritus consistently reduced at every successive assessment in all groups (Table 4). The difference in reduction of pain among the three groups was not significant. Reduction in pruritus, however, was significantly faster with 5FU than the other groups.

**Table 4:** Mean pre-injection scores for subjective parameters

**Subjective parameter**	**Group**	**Mean Pre-injection Scores** *									
		**0w**	**3w**	**6w**	**9w**	**12w**	**15w**	**18w**	**21w**	**24w**	**30w**
Pain	TAC	2.05±0.89	1.8±0.70	1.2±0.62	0.65±0.59	0.35±0.59	0.15±0.49	0.05±0.22	0±0	0±0	0±0
	5FU	2.3±0.86	1.9±0.72	1.35±0.67	0.8±0.62	0.4±0.50	0.2±0.41	0.1±0.31	0±0	0±0	0±0
	T+F	2.55±0.69	2.25±0.79	1.6±0.75	1.05±0.83	0.6±0.75	0.4±0.50	0.2±0.41	0.1±0.31	0±0	0±0
Pruritus	TAC	2.75±0.44	2.75±0.44	2.5±0.61	2.15±0.59	1.75±0.64	1.3±0.66	1.05±0.69	0.65±0.59	0.35±0.49	0.30±0.45
	5FU	2.85±0.37	2.35.49	1.7±0.66	1.1±0.64	0.85±0.59	0.35±0.59	0.1±0.31	0.05±0.22	0.05±0.22	0.05±0.22
	T+F	2.7±0.47	2.55±0.51	2.2±0.52	1.65±0.67	1.25±0.72	0.9±0.64	0.55±0.60	0.3±0.57	0.2±0.52	0.2±0.52

* Values denoted as Mean±SD.

**Fig. 2:**
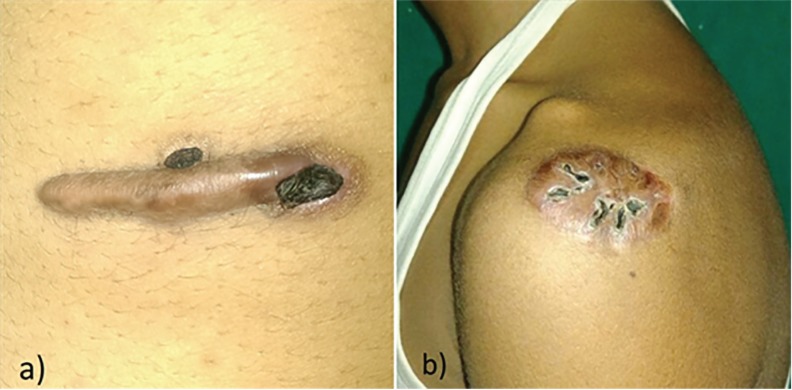
Ulceration following intralesional 5FU injection

Telangiectasias and skin atrophy were seen most frequently in TAC group. Skin ulceration was a common problem in 5FU group (Figure 2), less in T+F group and non-existent in TAC group. Systemic adverse effects in the form of anemia, leucopenia or thrombocytopenia were not noted in any patient. No other abnormalities were noted in any other blood investigations. A summary of adverse effects observed in all groups is summarized in Table 5. Pain at injection site was a common problem in 5FU group (140/166 injection episodes, 84%) compared to the TAC (42/170, 24%) and T+F (58/168, 34%) groups.

**Table 5:** Summary of adverse effects

**Adverse effect**	**Treatment Group** *		
	**TAC**	**5FU**	**T+F**
Telangiectasia	3	0	1
Skin atrophy	4	0	2
Skin ulceration	0	9	4
Systemic adverse effects	0	0	0

* Values denote number of patients.

## DISCUSSION

Alibert in 1806 first coined the word ‘keloid’ to illustrate the way the lesions invaded surrounding normal tissue. They are a cause of cosmetic, physical as well as psychological embarrassment to patients.^8^ Available literature provides a plethora of treatment options, none of which have been shown to be efficacious beyond doubt. Intralesional injection of triamcinolone acetonide (TAC) acts in multiple ways, some of which are by decreasing in fibroblast proliferation, increasing collagen disintegration, suppressing of inflammation and decreasing endothelial budding.^16^^-^^19^ Additionally, it has been noted to cause a significant reduction in the levels of alpha-1-antitrypsin and alpha-2-macroglobulin levels., A varying dose of 10-40 mg/ml has been described in literature. We chose a dose of 40 mg/ml for our study. The most frequent adverse effects linked to TAC are telangiectasia, skin atrophy and altered pigmentation. 

5-fluorouracil is an antimetabolite which interferes with ribonucleic acid (RNA) synthesis and inhibits fibroblast proliferation. It also has an inhibitory effect on TGF-β induced expression of the type I collagen gene. DNA and RNA synthesis are affected at several levels, including inhibition of thymidylate synthetase., 5FU can be administered intralesionally in a dose of 50 mg/ml and has shown favorable results. No systemic complications of 5-FU, such as anemia, leucopenia and thrombocytopenia have been reported, but the common locally encountered adverse effects include pain at injection site, ulceration, burning and hyperpigmentation.

As a combination, TAC has been added to 5FU in a ratio of 1:9 which translates to an absolute TAC concentration of 4 mg/ml.^26^^,^ This dose may not be sufficient by itself in scar regression but it most likely plays a different role by countering the adverse effects of 5FU by its anti-inflammatory nature. Consequently, it is hypothesized that the benefit of faster response of 5FU can be obtained with this combination while avoiding adverse effects of the individual drugs. 

There is no consensus regarding the treatment interval. TAC has been given with an interval varying from 2 to 6 weeks whereas regimens containing 5FU have been given more frequently ranging from weekly to 3-weekly injections.^8^^,-^^30^ But a recent systematic review suggests that there is no clear correlation of interval with outcome.^26^ We chose an interval of 3 weeks to account for all three regimens.

Authors have described various methods of achieving pain relief at the site of injection viz. lignocaine as a separate injection or mixed in the same injection. In our view, diluting the drug would require more volume for the same active dose. Our argument against the latter is that the separate injection increases volume of injection causing more stretch, more pain and theoretically causes blanching with a smaller active dose. These could compromise on outcome and comparison and, hence, for the sake of our study, drugs were used in their undiluted form without anesthetic injection. In this study, pain at the site of injection was a common problem with regimens containing 5FU which is consistent with other studies.^8^^,^^28^^-^^34^ Pain seemed to be blunted by addition of TAC to 5FU. Oral analgesics alone were given to all patients in the study. Tattooing of 5FU, topical TAC following ablative radiofrequency and ablative laser have been described as alternatives.^,^

The combination regimen has been proven to be better than TAC alone.^12^^,,^ A recent meta-analysis by Ren et al concluded that TAC+5FU is safer and more efficacious than TAC alone. Studies have also shown the effectiveness of the combination to be significantly better than 5FU.^30^^,^ But all these studies cannot be directly compared to each other due to lack of standardization. We have attempted standardizing the comparison by using an accepted scar assessment scale. Even so, these objective parameters are observer dependent and prone to errors. Evaluation by the same independent trained observers, such as in our study, can help minimize this error. We also added a dimension of subjective assessment since keloids are ultimately more of a subjective concern for the patient.

In our study, although each regimen was more effective in one parameter than the other, the combination fared better overall, which is in line with the aforementioned studies. A lower number of adverse effects were observed when drugs were used as a combination. No systemic adverse effects of 5FU were noted in the study. A limitation of the current study is the short duration of follow-up. All patients in our study were observed for 30 weeks, during which there was no recurrence. A long term follow-up in such a prospective study is difficult; our interaction with such patients leads us to believe that this is probably because the patient is unwilling to return when he is convinced that his ‘disease’ has been apparently ‘cured’. Perhaps a longer prospective study focusing on recurrence might prove more useful in this regard. 

Triamcinolone acetonide, 5-fluorouracil and their combination are all effective in keloid scars. A combination of TAC+5FU seems to offer the balanced benefit of faster and more efficacious response with lesser adverse effects when compared to individual drugs. Treatment has to be individualized and can be combined with one or more modalities to aim for better efficacy and safety.

## CONFLICT OF INTEREST

The authors declare no conflict of interest.

## References

[1] Andrews JP, Marttala J, Macarak E, Rosenbloom J, Uitto J (2016). Keloids: The paradigm of skin fibrosis - Pathomechanisms and treatment. Matrix Biol.

[2] Kelly AP (2004). Medical and surgical therapies for keloids. Dermatol Ther.

[3] English RS, Shenefelt PD (1999). Keloids and hypertrophic scars. Dermatol Surg.

[4] Griffith SH (1966). Treatment of keloids with triamcinolone acetonide. Plast Reconstr Surg.

[5] Ketchum ID, Robinson DW, Masters FW (1971). Follow up on treatment of hypertrophic scars and keloids with triamcinolone. Plast Reconstr Surg.

[6] Roques C, Téot L (2008). The use of corticosteroids to treat keloids: a review. Int J Lower Extremity Wounds.

[7] Niessen FB, Spauwen PH, Schalkwijk J (1999). On the nature of hypertrophic scars and keloids: a review. Plast Reconstr Surg.

[8] Fitzpatrick RE (1999). Treatment of inflamed hypertrophic scars using intralesional 5- FU. Dermatol Surg.

[9] Wang XQ, Liu YK, Qing C, Lu SL (2009). A review of the effectiveness of antimitotic drug injections for hypertrophic scars and keloids. Ann Plast Surg.

[10] Shah VV, Aldahan AS, Mlacker S, Alsaidan M, Samarkandy S, Nouri K (2016). 5-Fluorouracil in the Treatment of Keloids and Hypertrophic Scars: A Comprehensive Review of the Literature. Dermatol Ther.

[11] Truong PT, Abnousi F, Yong CM, Hayashi A, Runkel JA, Phillips T, Olivotto IA (2005). Standardized assessment of breast cancer surgical scars integrating the Vancouver Scar Scale, Short-Form McGill Pain Questionnaire, and patients’ perspectives. Plast Reconstr Surg.

[12] Murray JC, Pollack SV, Pinnell SR (1981). Keloids: a review. J Am Acad Dermatol.

[13] Hochman B, Locali RF, Matsuoka PK, Ferreira LM (2008). Intralesional triamcinolone acetonide for keloid treatment: a systematic review. Aesth Plast Surg.

[14] Leventhal D, Furr M, Reiter D (2006). Treatment of keloids and hypertrophic scars: a meta-analysis and review of the literature. Arch Facial Plast Surg.

[15] Donkor P (2007). Head and neck keloid: treatment by core excision and delayed intralesional injection of steroid. J Oral Maxillofac Surg.

[16] Campaner AB, Ferreira LM, Gragnani A, Bruder JM, Cusick JL, Morgan JR (2006). Upregulation of TGF-beta1 expression may be necessary but is not sufficient for excessive scarring. J Invest Dermatol.

[17] Lee SS, Yosipovitch G, Chan YH, Goh CL (2004). Pruritus, pain, and small nerve fiber function in keloids: a controlled study. J Am Acad Dermatol.

[18] Urioste SS, Arndt KA, Dover JS (1999). Keloids and hypertrophic scars: Review and treatment strategies. Semin Cutan Med Surg.

[19] Wendling J, Marchand A, Mauviel A, Verrecchia F (2003). 5-fluorouracil blocks transforming growth factor-beta-induced alpha 2 type I collagen gene (COL1A2) expression in human fibroblasts via c-Jun NH2-terminal kinase/activator protein-1 activation. Mol Pharmacol.

[20] Bulstrode NW, Mudera V, McGrouther DA (2005). 5-fluorouracil selectively inhibits collagen synthesis. Plast Reconstr Surg.

[21] Ghoshal K, Jacob ST (1997). An alternative molecular mechanism of action of 5- fluorouracil, a potent anticancer drug. Biochem Pharmacol.

[22] Bijlard E, Steltenpool S, Niessen FB (2015). Intralesional 5-fluorouracil in keloid treatment: a systematic review. Acta Derm Venereol.

[23] Huang L, Cai YJ, Lung I, Leung BC, Burd A (2013). A study of the combination of triamcinolone and 5-fluorouracil in modulating keloid fibroblasts in vitro. J Plast Reconstr Aesthet Surg.

[24] Gupta S, Kalra A (2002). Efficacy and safety of intralesional 5-fluorouracil in the treatment of keloids. Dermatology.

[25] Manuskiatti W, Fitzpatrick RE (2002). Treatment response of keloidal and hypertrophic sternotomy scars: Comparison among intralesional corticosteroid, 5-fluorouracil, and 585-nm flashlamp-pumped pulsed-dye laser treatments. Arch Dermatol.

[26] Sharma S, Bassi R, Gupta A (2012). Treatment of small keloids with intralesional 5-fluorouracil alone vs intralesional triamcinolone acetonide with 5-fluorouracil. J Pak Assoc Dermatol.

[27] Kontochristopoulos G, Stefanaki C, Panagiotopoulos A, Stefanaki K, Argyrakos T, Petridis A, Katsambas A (2005). Intralesional 5- fluorouracil in the treatment of keloids: an open clinical and histopathologic study. J Am Acad Dermatol.

[28] Nanda S, Reddy BS (2004). Intralesional 5- fluorouracil as a treatment modality of keloids. Dermatol Surg.

[29] Issa MC, Kassuga LE, Chevrand NS, Pires MT (2013). Topical delivery of triamcinolone via skin pretreated with ablative radiofrequency: a new method in hypertrophic scar treatment. Int J Dermatol.

[30] Cavalié M, Sillard L, Montaudié H, Bahadoran P, Lacour JP, Passeron T (2015). Treatment of keloids with laser-assisted topical steroid delivery: a retrospective study of 23 cases. Dermatol Ther.

[31] Darougheh A, Asilian A, Shariati F (2009). Intralesional triamcinolone alone or in combination with 5-fluorouracil for the treatment of keloid and hypertrophic scars. Clin Exp Dermatol.

[32] Nagarur K, Raja N (2016). A comparative study between intralesional 5-fluorouracil combined with triamcinolone acetonide and triamcinolone acetonide alone in the treatment of keloids. Int J Basic Clin Pharmacol.

[33] Ren Y, Zhou X, Wei Z, Lin W, Fan B, Feng S (2017). Efficacy and safety of triamcinolone acetonide alone and in combination with 5-fluorouracil for treating hypertrophic scars and keloids: a systematic review and meta-analysis. Int Wound J.

[34] Ying ZZ (2007). Therapy function of 5-Fu associate with steroid to keloid (Medical Science) In Chinese. J Tongji Univ.

